# Rheological and Thermal Study about the Gelatinization of Different Starches (Potato, Wheat and Waxy) in Blend with Cellulose Nanocrystals

**DOI:** 10.3390/polym14081560

**Published:** 2022-04-11

**Authors:** Josefina Chipón, Kassandra Ramírez, José Morales, Paulo Díaz-Calderón

**Affiliations:** 1 Escuela de Nutrición y Dietética, Facultad de Medicina, Universidad de los Andes, Chile. Av. Monseñor Alvaro del Portillo Nº12.455, Las Condes, Santiago 7620001, Chile; jtchipon@miuandes.cl (J.C.); krramirez@miuandes.cl (K.R.); 2 Biopolymer Research & Engineering Laboratory (BIOPREL), Escuela de Nutrición y Dietética, Facultad de Medicina, Universidad de los Andes, Chile. Av. Monseñor Alvaro del Portillo Nº12.455, Las Condes, Santiago 7620001, Chile; jose.morales@miuandes.cl; 3 Centro de Investigación e Innovación Biomédica (CIIB), Facultad de Medicina, Universidad de los Andes, Chile. Av. Monseñor Alvaro del Portillo Nº12.455, Las Condes, Santiago 7620001, Chile

**Keywords:** gelatinization, cellulose nanocrystals, rheology, calorimetry

## Abstract

The goal of this work was to analyze the effect of CNCs on the gelatinization of different starches (potato, wheat and waxy maize) through the characterization of the rheological and thermal properties of starch–CNC blends. CNCs were blended with different starches, adding CNCs at concentrations of 0, 2, 6 and 10% *w/w*. Starch–CNC blends were processed by rapid visco-analysis (RVA) and cooled to 70 °C. Pasting parameters such as pasting temperature, peak, hold and breakdown viscosity were assessed. After RVA testing, starch–CNC blends were immediately analyzed by rotational and dynamic rheology at 70 °C. Gelatinization temperature and enthalpy were assessed by differential scanning calorimetry. Our results suggest that CNCs modify the starch gelatinization but that this behavior depends on the starch origin. In potato starch, CNCs promoted a less organized structure after gelatinization which would allow a higher interaction amylose–CNC. However, this behavior was not observed in wheat and waxy maize starch. Insights focusing on the role of CNC on gelatinization yielded relevant information for better understanding the structural changes that take place on starch during storage, which are closely related with starch retrogradation. This insight can be used as an input for the tailored design of novel materials oriented towards different technological applications.

## 1. Introduction

Starch and cellulose are the most widely distributed polymers in nature. Starch is found in the form of granules which are energy reservoirs for plants [1]. Cellulose, in turn, is part of the structural basis of cell walls in plant tissues, normally forming complexes with hemicellulose and lignin [2]. Although starch and cellulose have been widely used in food science and technology, in recent years, the interest in exploring the novel applications of these polymers and their composites has increased across completely different fields. Indeed, the literature has reported the use of cellulose as a strategy to improve some poor physical properties of starch such as brittleness, low mechanical resistance, high gas permeability and high hygroscopicity [3,4]. This strategy can be useful for the tailored design of starch–cellulose composites destined for food packaging and coating materials [4,5,6,7], scaffolds for tissue engineering (e.g., wound healing) [8,9,10] as well as applications in bioengineering and the pharmaceutical industry [3,8,11]. 

Likewise, more recently, interest in exploring the use of nanocellulose in the design of starch-based composites has also increased. Among the more interesting choices are cellulose nanocrystals (CNCs). CNCs correspond to the crystalline fractions presented in the elemental fiber of a cellulose microfibril [12,13]. CNCs are normally produced by acid hydrolysis under severe conditions in terms of concentration and temperature, which allows obtaining nanosized crystalline whiskers [14,15]. Depending on hydrolysis conditions and the cellulose source, CNCs have dimensions of approximately 120–200 nm in length and have a negative charge (z-potential lower than −30 mV) [14,15].

Despite the effect of CNCs on the physical properties and functionality of different starches being described in the literature, studies describing the role of CNCs during the gelatinization of starch are scarce. The gelatinization of starch has been well-described in terms of the mechanism of granule swelling, granule disruption, the loss of birefringence and amylose and amylopectin leaching due to the effect of temperature and stirring [16,17,18]. However, a comprehensive study of the gelatinization of starch in the presence of nanocrystals will help to understand the behavior of starch during the storage when the complex process of self-association and self-assembling will take place. This behavior explains the retrogradation of starch.

Therefore, this work aimed to study the gelatinization of starches from different sources (potato, wheat and waxy maize) in the presence of CNCs produced from cotton cellulose pulp. Pasting parameters closely related to starch gelatinization (such as pasting temperature, peak viscosity, hold viscosity and breakdown viscosity) were assessed in starch–CNC blends by rapid visco-analysis (RVA). The viscoelasticity of these blends were evaluated by rheology test at 70 °C, whereas the gelatinization temperature and enthalpy were assessed by differential scanning calorimetry (DSC). 

## 2. Materials and Methods

### 2.1. Materials

Native potato (Avebe, Veendam, The Netherlands), wheat (Sigma Aldrich, Darmstadt, Germany) and waxy maize (Sigma Aldrich, Darmstadt, Germany) starches were purchased in powder form. Cellulose nanocrystals (CNCs) produced from cotton fibers were purchased in freeze-dried form from The Process Development Center of University of Maine (Orono, ME, USA). Starch and CNCs were used as received without further purification and stored at room temperature until further use.

### 2.2. Methods

#### 2.2.1. Sample Preparation

CNC suspensions were prepared for blending with native starch to have a starch suspension with an CNC concentration of 0, 2, 6 and 10% *w/w* dry cellulose (Equation (1)). For this purpose, well-defined amounts of CNCs were suspended in distilled water (conductivity < 10 mS) only by stirring. Before being mixed with starch, CNCs were sonicated (1500 W, 5 min) at ambient temperature (20 ± 2 °C) to promote the complete dispersion of nanocrystals:

CNC concentration (%, *w/w*) = (CNC_weight_/(CNC_weight_ + Starch_weight_)) × 100 (1)

#### 2.2.2. Gelatinization of Starch–CNC Blends by Rapid Visco-Analysis (RVA)

The gelatinization of starch–CNC blends was carried out by rapid visco-analysis (RVA 4500, Perten Instruments, North Ryde, Australia) in accordance with the methodology proposed by Diaz-Calderon et al. [19] with minor modifications. Two grams of native starch was weighed in aluminum canisters and 25 mL of CNC suspension with different cellulose concentration was transferred using a micropipette. The canister was then inserted into the instrument and viscosity patterns were obtained as a function of temperature: holding at 25 °C during 2 min; heating between 25 and 95 °C at 13.5 °C/min; holding at 95 °C for 5 min; cooling to 25 °C at 13.5 °C/min; and holding at 25 °C for 1 min. The analysis was performed under constant stirring (160 rpm). As the goal of this work was focused on understanding the gelatinization of native starch, the pasting parameters evaluated were pasting temperature (°C), peak viscosity (cP), hold viscosity (cP) and breakdown viscosity (difference between hold viscosity and peak viscosity, cP). All measurements were carried out at least in triplicate. 

#### 2.2.3. Rheological Characterization of Gelatinized Starch–CNC Blends

The changes in viscoelasticity of starch–CNC blends were carried out by rheology (Discovery HR-2, TA Instrument, New Castle, DE, USA). Samples analyzed by rheology were prepared using the RVA machine and following the same protocol explained in Section 2.2.2, but with the cooling stage finishing at 70 °C. Once the protocol was finished, the RVA canister was immediately transferred to a controlled bath set at 70 °C. Samples were collected from the canister and transferred to the rheometer. 

Apparent viscosity (Pa·s) and shear stress (Pa) were assessed from the rotational flow curve obtained at 70 °C using cone geometry (stainless steel, 40 mm diameter, 0:30:7 angle and 15 μm truncation) in the shear rate range between 1 and 1000 1/s.

Dynamic changes in storage modulus (G′, Pa) and loss modulus (G″, Pa) were obtained through a frequency sweep carried out at 70 °C to analyze the behavior of G′, G″, and the loss factor (G″/G′) as a function of angular frequency from 1 to 648 rad/s at 0.5% strain which was within the linear viscoelastic range (LVR) previously defined by an amplitude sweep (0.1–1000%) at 70 °C. A flat plate geometry (5 cm diameter) was used for analysis and a 300-micron gap was selected for testing. The analysis considered at least five replicates per experimental condition. 

#### 2.2.4. Thermal Properties of Gelatinized Starch–CNC Blends

Gelatinization temperature (°C) and enthalpy (J/g starch) were measured by differential scanning calorimetry (DSC 1, Mettler-Toledo, Greinfensee, Switzerland) following the protocol reported by Díaz-Calderón et al. [19]. In order to improve the resolution signal, a higher concentration of the starch weight suspensions was used, keeping the CNC weight previously indicated fractions (0–10% *w/w*). The starch concentration was 20% *w/v*. Approximately 60 µL of starch–CNC blend suspensions were loaded into 100 µL aluminum pans and then hermetically sealed. The DSC was calibrated using indium (melting temperature and enthalpy of 156.5 ± 1.56 °C, H = 28.6 ± 1 J/g), and an empty pan was used as a reference. Thermal properties of the suspensions were measured as follows: holding temperature at 5 °C during 3 min, heating from 5 °C to 85 °C at 10 °C/min, and holding at 85 °C during 3 min. The gelatinization temperature (°C) was recorded from the onset of the endothermic peak associated with the starch granule swelling, while gelatinization enthalpy was considered as the area under the endothermic peak. Gelatinization enthalpy was normalized in terms of starch dry mass and was expressed in J/g starch. All measurements were performed at least in triplicate.

#### 2.2.5. Statistical Analysis

Where appropriate, the statistical significance was assessed by a paired *t*-test (same variances) and ANOVA using the Solver tool in Excel (Office 2016, Microsoft Corp., Redmond, WA, USA). 

## 3. Results

### 3.1. Gelatinization of Starch–CNC Blends by Rapid Visco-Analysis (RVA)

**Figure 1:**
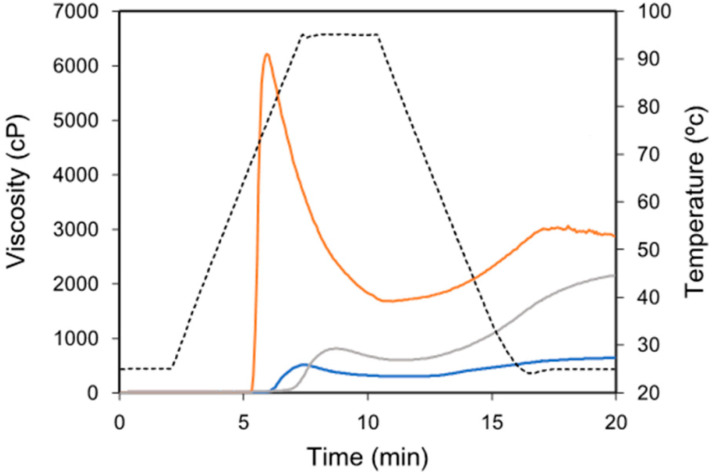
RVA profiles in starch from the different sources: potato (orange line), wheat (grey line) and waxy maize (blue line). Dotted line represents the temperature profile used during analysis.

Viscosity patterns of starch samples resulted from RVA are presented in Figure 1. As expected, the viscosity pattern showed marked differences among the starches tested: potato, wheat and waxy maize. From the viscosity patterns, the pasting parameters that allowed characterizing the gelatinization of starch were assessed, which in our study were pasting temperature, peak viscosity, hold viscosity and breakdown viscosity. The assessments of the pasting parameters for our starch–CNC blends are presented in Table 1.

Significant differences (*p*-value < 0.05) in the pasting parameters were observed among the pure starch samples (0%CNC). Regarding the pasting parameters of starch–CNC blends, our results show that the presence of CNC did not produce a significant difference (*p*-value > 0.05) on the pasting temperature. However, CNC produced a significant decrease (*p*-value < 0.05) in peak viscosity which was proportional to the increase in CNC concentration only in potato starch. In wheat and waxy starch, a decrease in peak viscosity was only detected at the highest CNC concentration tested. On the other hand, hold viscosity (also called through viscosity or hot-paste viscosity [16]) was significant lower (*p*-value < 0.05) only at the highest CNC concentration tested (10%) in potato and waxy maize starch. This parameter was not significant modified (*p*-value > 0.05) in the wheat starch blend. Likewise, the breakdown viscosity was significantly reduced (*p*-value < 0.05) by CNC in potato starch, and only at the highest CNC level tested in wheat and waxy starch. Interestingly, the assessment of the relative decrease only showed a marked decrease in potato starch, whereas in wheat and waxy starch, this ratio was lower only at the highest CNC level tested. 

### 3.2. Rheological Characterization of Gelatinized Starch–CNC Blends

**Figure 2:**
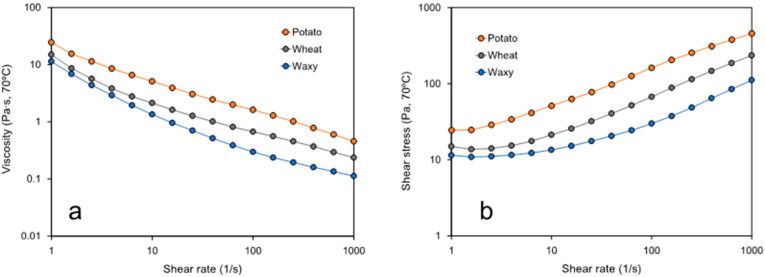
Flow curve in gelatinized pure starch samples (0%CNC) at 70 °C: (**a**) apparent viscosity and (**b**) shear stress as a function of shear rate.

**Figure 3:**
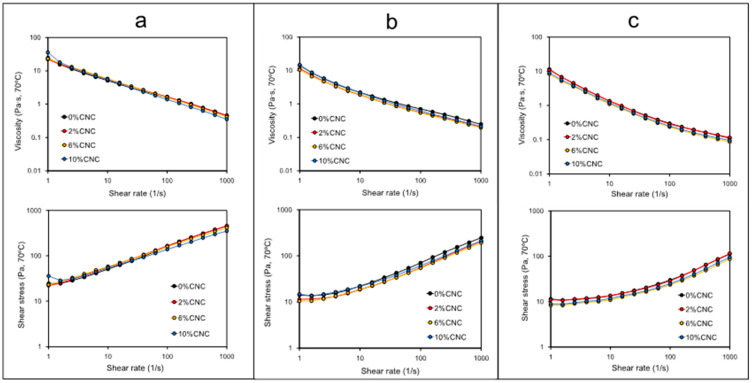
Flow curve in gelatinized starch samples at 70 °C and at different concentrations of CNCs: (**a**) potato; (**b**) wheat; and (**c**) waxy maize. Top plots correspond to apparent viscosity as a function of shear rate, whereas bottom plots correspond to shear stress as a function of shear rate.

Rheology analysis by rotational and dynamic tests were carried out at 70 °C on samples completely gelatinized by RVA. Apparent viscosity and shear stress as a function of shear rate recorded in pure starch samples and starch–CNC blends are presented in Figure 2 and Figure 3, respectively. In agreement with the hold viscosity data assessed by RVA (Table 1), the flow curve showed significantly higher viscosity values in potato starch at 70 °C over the complete shear rate range tested, whereas the waxy starch showed the lowest viscosity at 70 °C. Independent of the source, all starches showed non-Newtonian behavior. 

Figure 3 shows the effect of different concentrations of CNC on the apparent viscosity and shear stress as a function of shear rate tested at 70 °C in complete gelatinized starches. Our results show that CNC produced only slight changes in apparent viscosity and shear stress. Indeed, well-defined viscosity data recorded at 40 1/s (Table 2) showed that the presence of CNC only significant modified (*p*-value < 0.05) the apparent viscosity of waxy starch at 6% and 10%, tested at 70 °C. Likewise, the non-Newtonian condition of gelatinized starches tested at 70 °C was not modified by the presence of CNC, independently of the starch source. As expected, and following the same trend observed in Figure 3, gelatinized potato starch–CNC blends showed the highest apparent viscosity values, whereas waxy maize–CNC showed the lowest values of viscosity, independently of the CNC concentration. The same behavior was observed in terms of shear stress as a function of shear rate in the starch–CNC blends. 

**Figure 4:**
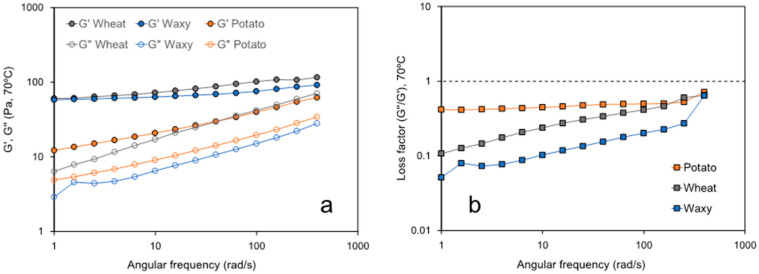
G′ and G″ (**a**) and loss factor (G″/G′) (**b**) assessed as a function of angular frequency in gelatinized starch samples at 70 °C.

**Figure 5:**
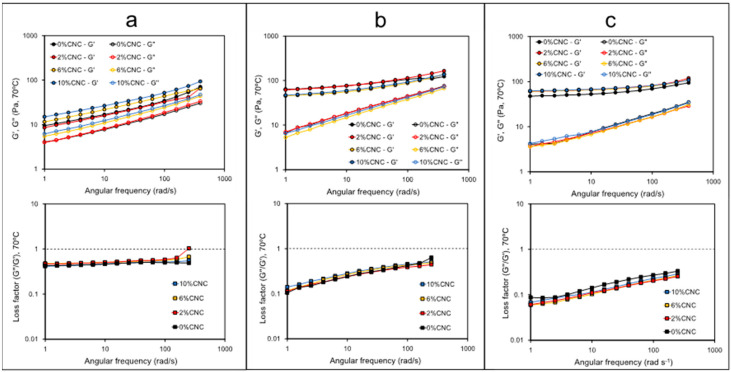
G′, G″ and loss factor (G″/G′) assessed as a function of angular frequency in gelatinized starch samples at 70 °C and at different concentrations of CNCs: (**a**) potato; (**b**) wheat; and (**c**) waxy maize. Top plots correspond to a frequency sweep as a function of shear rate, whereas the bottom plots correspond to the loss factor as a function of shear rate.

The viscoelastic characterization of starch–CNC blends is presented in Figure 4 and Figure 5. Dynamic tests were carried out within the linear viscoelastic range previously defined by an amplitude sweep at 70 °C (Figures S1 and S2, Supplementary File). Wheat and waxy starch did not show significant differences (*p*-value > 0.05) in G′ and both showed a slight slope over the angular frequency range tested at 70 °C (Figure 4a). Potato starch showed a significantly lower G′ over the whole angular frequency tested along with a marked slope depicting the strong dependence of G′ with the angular frequency. All the gelatinized starch samples showed significantly higher values of G′ compared to values of G″, which represent the gel-like condition of starch blends after processing by RVA. The latter is also confirmed from the values of the loss factor at 70 °C which resulted in values lower than 1 in all samples tested (Figure 4b). However, at angular frequencies lower than 100 rad/s, the loss factor in potato starch was constant and with higher values than those assessed in the wheat and waxy starch. 

The effect of CNC on the viscoelasticity of our starch blends is shown in Figure 5, and selected values of G′, G″ and the loss factor assessed at 10 rad/s and 70 °C are presented in Table 3. Our results show that the effect of CNC on the G′ is different depending on the starch source. Thus, CNC produced a significant increase in G′ in potato starch and waxy starch (*p*-value < 0.05) but a significant decrease in wheat starch. However, this effect was not proportional to the CNC concentration (Table 3). Similar behavior was observed with G″ data. The behavior of G′ and G″ were well reflected by the loss factor data which increased in the case of potato and wheat starch as a function of CNC concentration and decreased in waxy starch in the presence of CNC. 

### 3.3. Thermal Properties of Gelatinized Starch–CNC Blends

**Figure 6:**
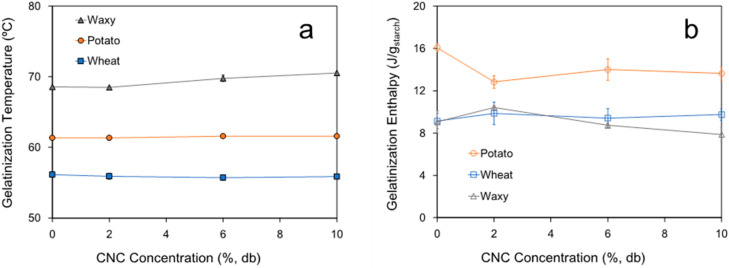
Gelatinization temperature (**a**) and enthalpy (**b**) of starch from different sources (potato, wheat and waxy) as a function of CNC concentration. Continuous lines only correspond to guide the eye.

Both the gelatinization temperature (°C) and gelatinization enthalpy (J/g starch) of starch–CNC blends are shown in Figure 6. The presence of CNC had little effect on the gelatinization temperature recorded from the onset of the endothermic peak associated with starch granule swelling, which agrees with the behavior of pasting temperature assessed by RVA (Table 1). As far as enthalpy is concerned, significant changes were only observed in potato starch, where the presence of CNC produced a decrease in enthalpy but without significant differences (*p*-value > 0.05) among CNC concentrations. In both wheat and waxy starch, the presence of CNC did not change the value of energy necessary to trigger the granule swelling and disruption. 

## 4. Discussion

Differences in the viscosity patterns among starches showed in Figure 1 can be explained by polymorphism expressed by each starch (e.g., A-type in case of wheat and waxy, B-type for potato), which are dependent on the source, the hierarchical structure and lamellar organization in native granules and the branch-chain length of amylopectin [20]. On the other hand, the assessment of pasting parameters (Table 1) can be used as an approach to understand the effect of CNCs on the gelatinization of starch. Thus, the decrease in peak viscosity suggests that the mechanism of granule swelling and disruption is affected by the presence of CNCs, although this behavior would be dependent of the starch source. The literature has only partially reported the effect of CNCs (or other nanowhiskers) on the pasting properties of gelatinized starch, but with contradictory results. For instance, Cui et al. [21] described that CNCs produced a significant increase in peak viscosity in both waxy maize and sweet potato starch, which was attributed to a cross-linking effect via hydrogen bonding between CNCs and starch granules, resulting in a reduced degree of granule swelling. However, Ji et al. [22] found that chitin nanowhiskers significantly reduced the peak viscosity in potato starch, explaining this behavior by an effect of replacement of starch with chitin nanowhiskers causing a dilution of the concentration of starch, and decreasing the peak viscosity. George et al. [23] recently suggested that a lower hydration property restricts the granule swelling and thereby reduces the peak viscosity. Likewise, the literature hypothesized about the ability of cellulose particles to bind water or compete for free water with leached amylose and ungelatinized granules affecting hydration, mobility and granule swelling [19,24]. This could be another reason why in our study the presence of CNCs reduced the peak viscosity. 

On the other hand, the pasting temperature was not changed by CNCs, suggesting that the process by which the starch granules begin to swell is not modified by the presence of CNCs, regardless of the starch origin. These results agree with the study of Cui et al. [21] who reported that the presence of CNCs did not modify the pasting temperature in normal maize, waxy maize and sweet potato starches. However, that study was carried out using samples with marked differences in starch concentration, which resulted in values of the pasting temperature which were identical between waxy and potato. Previously, Ji et al. [22] reported that chitin-based nanowhiskers did not change the pasting temperature of maize starch. Most recently, George et al. [23] reported that the addition of starch nanocrystals in concentrations of up to 15% did not produce significant changes in the pasting temperature of rice and wheat starch. 

The hold viscosity was shown to be significantly lower at the highest CNC concentration tested in potato and waxy starch, which agree with the data of apparent viscosity (Table 2). The literature lacks works that describe changes in viscosity in gelatinized starch by the effect of nanoparticles and nanocrystals. BeMiller [25] reviewed the effect of different hydrocolloids on the pasting, paste and gel properties of different starch–hydrocolloids composites, finding that changes in viscosity in gelatinized starches would be in dependent of the hydrocolloid molecular size, and concluding a possible direct correlation between the molecular weight and increase in viscosity. Most recently, Ji et al. [22] reported that the presence of chitin nanowhiskers did not modify the hold viscosity in maize and potato starch pastes, which was explained by a possible effect of the interaction between chitin nanowhiskers and starch molecules via hydrogen bonds among the OH groups of nanowhiskers and starches. Likewise, the literature reported that a significant decrease in breakdown viscosity would suggest a reduced capacity for the swelling of starch granules [26]. This could be the case in the data reported in Table 1; however, the evaluation of the relative decrease in viscosity during gelatinization resulted in potato starch showing a decrease from values of 3.80 to 1.26 by the effect of CNCs (Table 1). In the case of wheat and waxy maize starch, the values of relative decrease remained constants except for the highest CNC concentration, which was lower in both cases. These results support the fact that CNCs mainly modify the RVA pattern and pasting properties in potato starch. 

Regarding the apparent viscosity behavior of starch at 70 °C, our results show a significant higher viscosity in potato starch (Figure 2). This behavior was correlated with the substantially longer branch-chains length of the potato starch which once leached out during gelatinization to offer higher resistance to flow [20]. The non-Newtonian behavior of starchy pastes can be explained by the alignment effect of starchy particles in the direction of the shear during the analysis, as has been reported in the literature [20,27,28,29]. The increase in shear stress as a function of the shear rate (Figure 2b) associated with changes in viscosity has been related with the solid–liquid character of the gelatinized suspensions [30], as well with the increase in interaction between starch particles and fibrils. Agglomerates or network structures able to be broken or aligned during rheological measurements result in higher shear stress values [24,27]. 

Changes in pasting properties can be better understood through the viscoelastic characterization of starch pastes. The results shown in Figure 4 suggest that once gelatinized, potato starch shows a less ordered structure (lower G′, less elastic) compared to wheat and waxy starch. In wheat and waxy, the starchy paste is more elastic at lower angular frequencies, suggesting the time-dependence condition of the structural organization of gelatinized starch tested at 70 °C. This behavior may help to explain the differences previously found in hold viscosity (Table 1) and apparent viscosity (Figure 3 and Figure 4). Since potato starch was shown to be less organized and less elastic once gelatinized, the viscosity in potato starch is higher than that observed in wheat and waxy starch, which is consistent with the higher values of the loss factor observed in the potato starch. These results suggest that the gelatinization mechanism could be different depending on the starch origin or could be influenced by the polymorph organization presented in the native granule. Ai and Jane [20] have reported that the integrity of a swollen granule after gelatinization is essential to understanding the formation of strong gels. The granules of potato starch readily swell and disperse during gelatinization because of the lack of amylose–lipid complex formation which helps to maintain the integrity of swollen starches [20]. Thus, this behavior agrees with results shown in Figure 5 and Table 3, where gelatinized potato starch showed a lower G′ and higher loss factor values at 70 °C. Similar behavior was reported by Cui et al. [21] whom despite testing the viscoelasticity of different gelatinized starch–CNC gels at 4 °C, also showed that sweet potato starch has a lower G′ and higher loss factor values compared to normal maize and waxy maize starch. 

Regarding the effect of CNCs on the viscoelasticity of gelatinized starches assessed at 70 °C, our results showed to be strongly dependent on the starch origin. Only a few studies reporting viscoelastic data in starch–CNC blends are available in the literature. For instance, Cui et al. [21] suggested the occurrence of interactions between CNCs and amylose, allowed by the high specific surface area and hydroxyl groups of CNCs. These interactions would explain why, in their study, CNCs delayed retrogradation in both sweet potato and normal maize starch. Our results show that waxy starch would retain structural order after gelatinization due to an increased G′ and decreased loss factor as a function of CNC concentration (Figure 5, Table 3). In turn, in both potato and wheat starch, the presence of CNCs increased the loss factor, suggesting a less organized structure [31]. Interestingly, CNCs decreased the value of G′ in wheat starch, whereas in potato starch, CNCs increased G′. However, the potato starch showed higher values of the loss factor compared with the wheat starch despite the fact that CNCs also lead to an increase in G″ in potato starch (Figure 5, Table 3). This behavior could be explained by the higher interaction amylose–CNC promoted by the leaching of amylose during gelatinization, which in the case of potato, is higher than in other starches, as was proposed by Ai and Jane [20]. Thus, the higher amylose–CNC interaction occurring in potato starch could explain the higher values of G′ and G″, which was not observed in wheat and waxy starches. Future studies of this point should include using IR-spectroscopy or RAMAN spectroscopy to characterize the type and intensity of these interactions.

Thermal characterization by DSC showed a good correlation with rheology data. The fact that the gelatinization temperature (Figure 6a) and pasting temperature (Table 1) of each starch did not show significant differences (*p* > 0.05) suggests that temperature related to the starting point of starch gelatinization is not modified by CNCs. Similar behavior was reported by Cui et al. [21] and Ji et al. [22]. Differences among the values of gelatinization temperature and pasting temperature could be explained because gelatinization temperature by DSC is detected from the point at which the double helices of amylose and amylopectin begin to unfold, whereas the pasting temperature from RVA is detected from the point at which the granule starts to swell [32]. On the other hand, differences detected between the gelatinization enthalpies of different starches also reflect differences in how these starches gelatinize. Thus, higher values of enthalpy in potato starch agree with the fast swelling and higher dispersion because of the absence of the amylose–lipid complex which helps to maintain the integrity of swollen starches [20]. However, the effect of CNCs reducing the value of the gelatinization enthalpy, which has been reported by other authors in the literature [21,33], can have more than one explanation. For example, Cui et al. [21] explained the reduction in enthalpy by the addition of CNCs due to an effect of the inhibition of gelatinization and conservation of double helices. Likewise, the ability of cellulose particles to bind water or compete for free water with leached amylose affecting hydration and granule swelling was hypothesized in the literature [19,26]. However, a potential effect of amylose–CNC interaction as has been suggested by our rheological characterization should not be neglected. Nonetheless, this behavior could help explain the lower enthalpy observed in wheat and waxy starch which were not modified by the presence of CNCs (Figure 6b). In this sense, as has been proposed in the previous section, a complementary analysis carried out by IR-spectroscopy and NMR could help understand this phenomenon that has not been extensively studied to date and may open up a new area of research.

## 5. Conclusions

Our results suggest that the presence of CNC produces changes in the mechanism of granule swelling and disruption during gelatinization, along with promoting a certain amylose–CNC interaction, although this behavior would be dependent of the starch origin. Thus, in potato starch, there was a marked decrease in peak viscosity and breakdown viscosity over CNC concentration, both values being consistent with the assessment of relative decrease in viscosity. This behavior was not observed in wheat starch and waxy starch. Hold viscosity measured by RVA agreed with apparent viscosity measured by rotative test, whereas both the peak temperature and gelatinization temperature were not changed by the presence of CNCs in all the starches studied. However, viscoelastic characterization at 70 °C showed higher values of loss factor in potato starch–CNC, suggesting a less organized structure after gelatinization, which in turn showed good correlation with the increase in G″ at different CNC concentration. Our results are presumably influenced by how potato starch gelatinizes and would not be affected by the presence/absence of amylose, as data from wheat and waxy starch suggest. The amylose–CNC interaction in potato starch could explain the higher values of enthalpy detected by calorimetry. However, complementary studies are needed to define the characteristics of these interactions. 

These results can be useful in different technological applications based on the use of starch–nanocellulose composites. For instance, for the design of novel composite materials for packaging, bioplastics, bioprinting and food ingredients, to name a few. The potential impact of these biomaterials on sustainable processes opens interesting perspectives in terms of industrial application. 

## Figures and Tables

**Table 1:** Pasting properties recorded during the gelatinization of starch (potato, wheat and waxy corn) assessed by RVA in starch–CNC blends. Values in brackets correspond to standard deviation. Different upper letters in the same column represent significant differences (*p*-value < 0.05). The relative decrease was defined as the ratio of peak viscosity over hold viscosity in starch samples containing the same amount of CNC.

	Pasting Temperature (°C)			Peak Viscosity (cP)			Hold Viscosity (cP)			Breakdown Viscosity (cP)			Relative Decrease		
CNC (%, db)	Potato	Wheat	Waxy	Potato	Wheat	Waxy	Potato	Wheat	Waxy	Potato	Wheat	Waxy	Potato	Wheat	Waxy
0	69.2 (0.5) ^a^	87.0 (0.1) ^a^	79.8 (1.1) ^a^	6217.6 (31.1) ^a^	810.1 (9.8) ^a^	514.2 (11.9) ^a^	1638.2 (40.3) ^a^	598.6 (8.4) ^a^	296.0 (7.0) ^a^	4579.4 (172.9) ^a^	211.4 (6.7) ^a^	218.2 (5.4) ^a^	3.80	1.35	1.74
2	69.1 (0.5) ^a^	87.0 (0.0) ^a^	79.9 (0.4) ^a^	3919.4 (98.3) ^c^	776.9 (15.8) ^b^	498.6 (9.2) ^a^	1615.4 (50.7) ^a^	562.0 (15.2) ^b^	287.4 (5.9) ^a^	2304.1 (55.9) ^c^	214.5 (3.2) ^a^	211.2 (3.6) ^a^	2.43	1.38	1.73
6	69.3 (0.5) ^a^	87.0 (0.1) ^a^	79.8 (0.4) ^a^	2667.4 (32.4) ^d^	770.8 (13.4) ^b^	438.2 (5.8) ^b^	1653.0 (15.9) ^a^	573.2 (14.9) ^a,b^	257.8 (3.8) ^b^	1014.4 (22.7) ^d^	197.6 (5.9) ^b^	180.4 (3.3) ^b^	1.61	1.34	1.70
10	68.9 (0.4) ^a^	87.1 (0.2) ^a^	80.3 (0.9) ^a^	1921.0 (14.9) ^e^	701.1 (73.8) ^c^	395.2 (6.4) ^c^	1526.6 (13.9) ^b^	578.8 (41.5) ^a,b^	245.6 (5.5) ^c^	394.4 (11.3) ^e^	122.2 (33.4) ^c^	149.6 (2.5) ^c^	1.26	1.21	1.61

**Table 2:** Apparent viscosity and shear rate of starch–CNC blends assessed at a shear rate of 40 1/s and 70 °C. Values in brackets correspond to standard deviation. Different upper letters in the same column represent significant differences (*p*-value < 0.05).

	Viscosity (Pa·s, 40 1/s, 70 °C)			Shear Stress (Pa·s, 40 1/s, 70 °C)		
CNC (%, db)	Potato	Wheat	Waxy	Potato	Wheat	Waxy
0	2.44 (0.08) ^a^	1.07 (0.07) ^a^	0.51 (0.05) ^a^	97.3 (1.5) ^a^	42.5 (2.9) ^a^	20.4 (1.4) ^a^
2	2.64 (0.05) ^b^	0.85 (0.02) ^b^	0.50 (0.03) ^a^	105.2 (2.1) ^b^	33.9 (1.0) ^b^	19.8 (1.3) ^a,c^
6	2.60 (0.03) ^b^	0.85 (0.03) ^b^	0.40 (0.02) ^b^	103.5 (1.3) ^b^	33.8 (1.3) ^b^	16.5 (1.4) ^b^
10	2.33 (0.06) ^a^	0.96 (0.09) ^a,b^	0.43 (0.01) ^b^	92.8 (8.1) ^a^	38.2 (4.4) ^a,b^	17.2 (1.0) ^b,c^

**Table 3:** G′, G″ and loss factor of starch–CNC blends assessed at an angular frequency of 10 rad/s and 70 °C. Values in brackets correspond to standard deviation. Different upper letters in the same column represent significant differences (*p*-value < 0.05).

	G′ (Pa, 10 rad/s, 70 °C)			G” (Pa, 10 rad/s, 70 °C)			Loss Factor (G″/G′, 10 rad/s, 70 °C)		
CNC (%, db)	Potato	Wheat	Waxy	Potato	Wheat	Waxy	Potato	Wheat	Waxy
0	16.74 (0.9) ^a^	76.43 (3.0) ^a^	52.90 (2.4) ^a^	7.76 (0.2) ^a^	18.32 (1.6) ^a^	7.59 (0.9) ^a^	0.46 (0.02) ^a^	0.24 (0.025) ^a^	0.14 (0.072) ^a^
2	15.92 (0.8) ^a^	76.17 (3.8) ^a^	66.28 (4.2) ^b^	8.06 (0.3) ^a^	18.33 (0.9) ^a^	7.30 (0.3) ^a^	0.51 (0.01) ^b^	0.24 (0.002) ^a^	0.11 (0.010) ^a^
6	21.65 (1.8) ^b^	55.91 (3.7) ^b^	65.64 (3.2) ^b^	10.92 (0.7) ^b^	14.82 (0.9) ^b^	6.72 (0.4) ^a^	0.51 (0.02) ^b^	0.27 (0.06) ^a^	0.10 (0.001) ^a^
10	26.45 (1.2) ^c^	59.26 (2.4) ^b^	67.45 (3.7) ^b^	12.28 (0.5) ^b^	16.63 (0.6) ^a,c^	7.69 (0.5) ^a^	0.46 (0.01) ^a^	0.28 (0.004) ^b^	0.11 (0.003) ^a^

## Data Availability

Our study do not report supporting results.

## Supplementary Materials

The following supporting information can be downloaded at: https://www.mdpi.com/article/10.3390/polym14081560/s1, Figure S1: G′ (a) and G″ (b) assessed as a function of oscillation strain in gelatinized starch samples at 70 °C. Figure S2: G′ and G″ assessed as a function of oscillation strain in gelatinized starch samples at 70 °C, and at different concentrations of CNC: (a) potato, (b) wheat and (c) waxy maize.
