# Supplementary material for: Rheological and Thermal Study about the Gelatinization of Different Starches (Potato, Wheat and Waxy) in Blend with Cellulose Nanocrystals
Source: Polymers (Basel). 2022 Apr 11;14(8):1560. doi: 10.3390/polym14081560 (PMC9025455; doi:10.3390/polym14081560)
Supplement: Supplementary file 1 [file polymers-14-01560-s001.zip › polymers-1663574-supplementary.pdf]

polymers

MDPI

*Supplementary Materials*

# Rheological and Thermal Study about the Gelatinization of Different Starches (Potato, Wheat and Waxy) in Blend with Cellulose Nanocrystals

**Josefina Chipón [1], Kassandra Ramírez [1], José Morales [2,3] and Paulo Díaz-Calderón [2,3,*]**

[1] Escuela de Nutrición y Dietética, Facultad de Medicina, Universidad de los Andes, Chile. Av. Monseñor Alvaro del Portillo Nº12.455, Las Condes, Santiago 7620001, Chile; jtchipon@miuandes.cl (J.C.); krramirez@miuandes.cl (K.R.)

[2] Biopolymer Research & Engineering Laboratory (BIOPREL), Escuela de Nutrición y Dietética, Facultad de Medicina, Universidad de los Andes, Chile. Av. Monseñor Alvaro del Portillo Nº12.455, Las Condes, Santiago 7620001, Chile; jose.morales@miuandes.cl

[3] Centro de Investigación e Innovación Biomédica (CIIB), Facultad de Medicina, Universidad de los Andes, Chile. Av. Monseñor Alvaro del Portillo Nº12.455, Las Condes, Santiago 7620001, Chile

* Correspondence: pdiaz@uandes.cl

## Figures

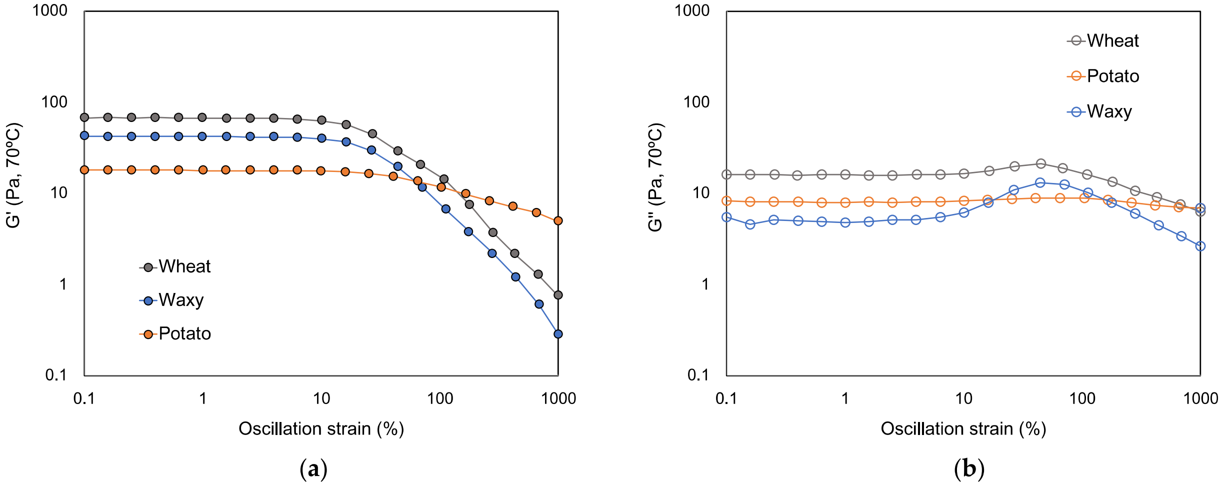


**Figure S1.** G′ (**a**) and G′′ (**b**) assessed as a function of oscillation strain in gelatinized starch samples at 70 °C.

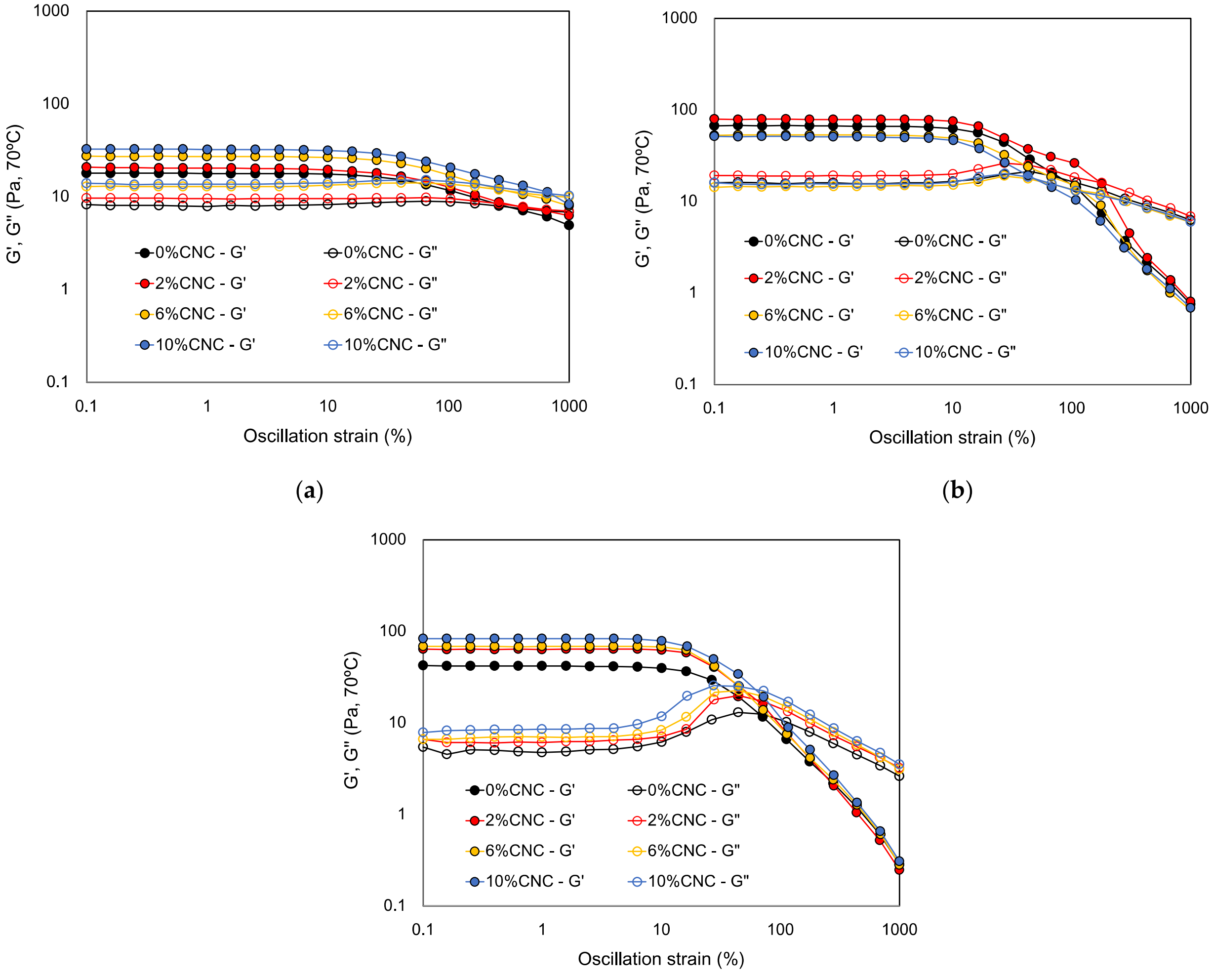


(**c**)

**Figure S2.** G′ and G′′ assessed as a function of oscillation strain in gelatinized starch samples at 70 °C, and at different concentrations of CNC: (**a**) potato, (**b**) wheat and (**c**) waxy maize.
